# Impact of religiosity on purchase intentions towards counterfeit products: investigating the mediating role of attitude and moderating role of hedonic benefits

**DOI:** 10.1016/j.heliyon.2021.e06026

**Published:** 2021-02-12

**Authors:** Azizul Yadi Yaakop, Hafiz Muhammad Hafeez, Malik Muhammad Faisal, Muhammad Munir, Majid Ali

**Affiliations:** a University Malaysia Terengganu, Malaysia; b University Institute of Management Sciences, PMAS, Arid Agriculture University, Rawalpindi, Pakistan; c Department of Management & Administrative Sciences, University of Narowal, Narowal, Pakistan; d Hailey College of Commerce, University of the Punjab, Lahore, Pakistan

**Keywords:** Religiosity, Intrinsic religiosity, Extrinsic religiosity, Counterfeit products, Purchase intentions, Hedonic benefit

## Abstract

This study was aimed at exploring the impact of religiosity on purchase intention towards counterfeit products by investigating the mediating role of consumer attitude. This study investigated religiosity as an independent variable, attitude towards counterfeit as a mediator while predicting the purchase intentions of the consumers. A self-administered questionnaire using a five-point Likert scale was used to collect data from the sample of 420 respondents who were from twin cities (Rawalpindi and Islamabad) of Pakistan. Structural equation modeling technique was used to achieve the set objectives. The results reveal a statistically significant effect of religiosity along with the significant mediating role of consumer attitude and the significant moderating role of hedonic benefits while predicting the purchase intentions of the consumers. This study also provides important insights for the researchers and the practitioners.

## 1 Introduction

In the previous studies, counterfeiting practices were categorically discouraged to safeguard the rights of the customers and consumers. Counterfeiting is a widespread dishonest market practice by the producers (Bush et al., 1989). The counterfeit products are inferior in quality and they are made or sold under some other famous brand names without seeking and attaining legal authorization (Chaudhry and Zimmerman, 2009). These products economically harm other corporations under the name of which counterfeit products are offered and sold. According to the published report (Connector Supplier, 2013; Economist Magazine, 2010; International Organization for Standardization (ISO), 2014), counterfeit products accounted for 5%–7% of total world trade in 2013 and they cost an estimated 2.5 million lost jobs worldwide in 2014. Interestingly, in 2013, around 5% of the products imported into the European Union were counterfeit products (OECD, 2016). According to the online report of International Chamber of Commerce (2020), in the year 2015, counterfeit products were having a worth of $1.7 trillion which was predicted to reach an amount of $4.2 trillion by 2022. Globally, counterfeit products are sold in an extensive range of products such as garments, technology, drinks, meals, medications, cigarettes, even though automobile and aircraft which need to be detected and punished (Berman, 2008). Religion is considered to be an extremely important matter for the majority of people around the world. It plays important role in guiding human behavior in all activities of his/her life (Zastrow and Kirst-Ashman, 2006). It is observed that some of the counterfeit products are offered with backup support by the religious attachments to the products. As it was established that religiosity and buying behaviors are significantly associated (Sood and Nasu, 1995), some corporations use it in their favor to form consumers' attitudes to buy counterfeit products. As religious believes, utilitarian values and attitudes significantly affect purchase intentions, they are tapped by the corporations. Previous studies highlighted the cognitive dissonance and attitude towards the creation of purchase intentions and ultimate buying behaviors of counterfeit products. In Pakistan, the sale of counterfeit products is on the rise since the majority of the people are highly religious. This fact is utilized by the corporations/producers as a strength to determine consumers'/customers' responses. Therefore, it becomes mandatory to study the consumer behaviors the consumers adopt while purchasing the products in connection with the religiosity and counterfeit products.

In past, fewer efforts have been completed over the strength of religiosity and this literature did not discover the association among the concentration of religiosity and buying behavior (purchase intention) purpose through exploratory the interceding part of attitudes towards Islamic and conservative conducts of ads and the subsequent attitude towards brands. Furthermore, not any of the literature completed contrast among Islamic and conservative conducts of advertisements. Nurhayati and Hendar (2019) recommended validating the effect of intrinsic religiosity on the purchase intentions of the consumers. Similarly, some other researchers (Mortimer et al., 2020) also recommended exploring this area to generate and advance the academic discussion. According to the facts, religious believes, utilitarian values, and attitudes significantly affect purchase intentions. The current literature is highly in the favor of cognitive dissonance because the attitude towards buying counterfeit products and the purchase intention in the religious perspective is highly related to the cognition involved with a religious person. Moreover, in Pakistan, religion is considered as a strength to determine consumers'/customers' response (Bupalan et al., 2019), it was recommended to study this area of research to investigate the potential effects of religion on the purchase intentions. Consequently, this study intended to discover whether or not religiosity had a positive effect on purchase intentions while considering the consumer attitude and hedonic benefits.

The current study was carried out to find the effects of intrinsic and extrinsic religiosity on purchase intention while considering the mediating role of attitude and moderating role of hedonic benefits towards the purchase intentions of counterfeit products. The current study added some important points that were helpful for academia and practitioners or professionals. This study provided the missing link of religiosity with the purchase intentions towards counterfeit products in the current era of e-commerce where counterfeit products are being offered (Bupalan et al., 2019; Mortimer et al., 2020; Nurhayati and Hendar, 2019).

## 2 Review of literature

### 2.1 Consumer religiosity

Intrinsic religiousness (referred to as intrinsic religiosity) is characterized as the ambitious inner motivation and love for religion that becomes the master motive of one's life (Allport, 1960). Intrinsic religiosity describes a person's knowledge and beliefs about religious norms, sources of well-being, gratification with life, struggles, and all other codes of life. According to the measurement of intrinsic religiosity is a matter of scientific investigation. He developed an Arabic version of Intrinsic Religiosity. He argued that giving Alms (zakat) provides intrinsic satisfaction (intrinsic religiosity) to the Muslims after which they feel serenity, harmony, cheerfulness, internal gratification, and a positive emotional state. Another recent study by Muthohar (2019) investigated the provision of Zakat as a source of intrinsic religiosity. Subsequently, intrinsic religiosity provides a shield against despair and dejections during tough times of life. According to the studies of Ardelt and Koenig (2009) and Foong et al. (2018), the sense of intrinsic religiosity encourages determination towards a sense of security and zeal to do good deeds in life. A recent study revealed that intrinsic religiosity and attitude towards purchasing counterfeit products are significantly associated (Jashim et al., 2020). Therefore, the following hypothesis was developed for testing:

H1 Intrinsic religiosity has a positive and significant relationship with attitude.

Extrinsic religiosity is an integral part of a religion that is revealed by the positive associations with others and the fulfillment of various practical obligations/actions. Several researchers have worked on this aspect of religiosity. According to Damayanti (2018), extrinsic religiosity is significantly associated with the religious benefits that are targeted to be achieved for the specific advantages. Research has been reporting a strong association of extrinsic religiosity with life satisfaction. Found a significant difference between religious and non-religious customers. The customers with higher extrinsic religiosity preferred to buy original products rather than counterfeit products. However, when they are deceived in terms of religious sentiments. In the study of Hwang (2018), it was revealed that there was a distinction between religious and non-religious customers. The religious customers showed a higher level of optimistic attitude while buying products. However, their lifestyles were simpler than non-religious customers who were pessimistic and had a superior lifestyle. Therefore, it was concluded that religious customers could be attracted with ease and their purchase intentions could be enhanced by including some religious aspect in some element of the products. According to Raggiotto et al. (2018), religiosity significantly affects the purchase intentions of the consumers or customers. They provided a religiosity framework where they treated it as an important antecedent of customer's buying behaviors. As consumer religiosity has a significant association with purchase intention and buying behavior, corporations embed religious elements in the advertisements to induce consumers' favorable buying behaviors (Nassè, 2020). In a recent study, Jashim et al. (2020) concluded that extrinsic religiosity and attitude towards purchasing counterfeit products were significantly associated. This means that both aspects of religiosity i.e. intrinsic religiosity and extrinsic religiosity play important role in framing the purchase intentions of the consumers. Therefore, the following hypothesis was developed for testing:

H2 Extrinsic religiosity has a positive and significant relationship with attitude.

### 2.2 Attitude towards counterfeit products

Attitude refers to the settled way of thinking or feeling. This study explores consumer's attitudes regarding buying counterfeit products by considering his/her knowledge and attitude towards original brands. Such comparison provides the consumers with a real comparative view of tangible (physical) and intangible characteristics of the products. A favorable comparison by the consumer is directly associated with the purchase intentions towards counterfeiting products and vice versa. Marticotte and Arcand (2017) argued that the consumer's attitude toward the original product and his/her attitude towards counterfeit products were significantly contributing to framing his/her purchase intentions. They also found that the mechanism of making a comparison had also created and induced the purchase intentions of the consumers regarding counterfeit products. Therefore, the following hypothesis was developed for testing:

H3 Hedonic benefit has a positive and significant relationship with purchase intentions.

Djuhardi and Kusumawati (2017) found that consumer's attitude towards counterfeit products was influenced by brand image, social affiliations, and personality which ultimately created purchase intentions of counterfeit sneakers of a famous brand. They concluded that consumers' attitude was significantly and positively contributing to the purchase intentions of counterfeit products due to much higher prices of original brands. Harun et al. (2020) argued that consumers continued to purchase counterfeit products due to their positive or favorable attitude towards counterfeit products in various aspects. They found a significant between consumer attitude and re-purchase activities of counterfeit products. Jashim et al. (2020) found a significant association between religiosity and attitude towards purchasing counterfeit products. So, religiosity is considered as an important factor that frames purchase intentions of counterfeit products by utilizing religious sentiments. Therefore, we conclude that attitude has a significant role in instigating or demoting buying behaviors and purchase intentions of counterfeit products. Therefore, the following hypothesis was developed for testing:

H4 Attitude has a positive and significant relationship with purchase intentions.

### 2.3 Purchase intention towards counterfeit products

Various studies have found significant results about purchase intentions of counterfeit products. The literature found various positive/favorable factors that induced consumers/customers to purchase counterfeit products. Hidayat and Diwasasri (2013) found that various social and personality factors of the respondents formed their purchase intentions towards counterfeit products. They argued that the consumers who had a positive attitude towards counterfeit products had more purchase intentions to buy counterfeit products. Koay (2018) found that the consumer who had a lack of a sense of responsibility was having higher purchase intentions towards counterfeit products. Bupalan et al. (2019) argued that markets were flooded with counterfeit products. As there were a lot of numbers of consumers who had purchase intentions, they were willing to buy counterfeit products. They explained the theory of planned behavior framework to depict consumers' purchase intentions towards counterfeit products. Bhatia (2018) found value consciousness, materialism, and social influence as the predictors of consumers' purchase intentions towards counterfeit fashion products. Therefore, it can be safely argued that positive factors/evaluations form purchase consumers' purchase intentions towards counterfeit products whereas negative factors/evaluations lower purchase consumers' purchase intentions towards counterfeit products. Therefore, the following hypotheses were developed for testing:

H5 Attitude mediates the relationship between intrinsic religiosity and purchase intentions.

H6 Attitude mediates the relationship between extrinsic religiosity and purchase intentions.

H7 Hedonic benefit moderates the relationship between attitude and purchase intentions.

**Figure:**
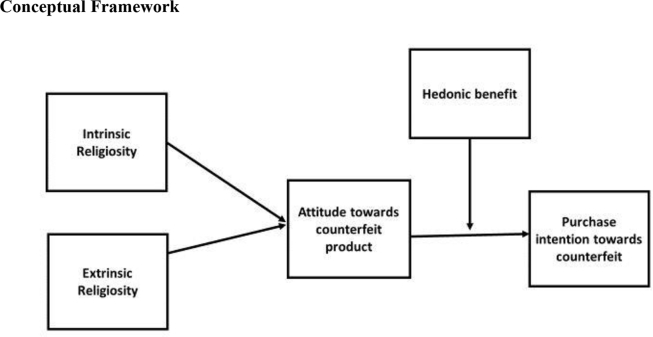

## 3 Methodology

The current study used a cross-sectional survey research design to achieve its research objectives. This research was based on two religiosity perspectives i.e. intrinsic religiosity and extrinsic religiosity for the individual unit of analysis (the consumers). The population of the current study included university students and employees who were believed to be potential consumers of counterfeit products. The students were enrolled in the universities of Rawalpindi and Islamabad and employees who were working in various organizations of Rawalpindi and Islamabad, Pakistan. A sample size of 384 was considered appropriate for the current study according to the confidence level of 95% and confidence interval (significance level) of 5% (Krejcie and Morgan, 1970; Munir and Azam, 2017; Munir et al., 2020). However, 600 questionnaires were distributed to counter the problem of a lower response rate. From the distributed questionnaires, 420 completely filled-in questionnaires were received.

With respect to the method of approaching the participants, findings, and arguments of business research methods were consulted. According to Sekaran and Bougie (2016), if a survey is confined to a local area, personally-administered questionnaires are deemed as an appropriate way of collecting the data. It has many advantages such as saving time, clarifying doubts of the respondents, opportunity to motivate the respondents to offer true answers, and bearing fewer expenses (Gnambs and Kaspar, 2015; Sekaran and Bougie, 2016). Therefore, the researchers chose the respondents who had the experience of purchasing counterfeit products.

All the variables included in the current study were measured by using already developed scales. The scale was used for the measurement of intrinsic religiosity was used. Extrinsic religiosity scale of Darvyri et al. (2014) having 7-items, the 5-items scale of the attitude of De Matos et al. (2007), 5-items scale of Yoo and Lee (2009) for hedonic benefits, and a 12-items scale of purchase intentions developed by Phau et al. (2009) was used. For data analysis, the current study used SPSS and AMOS software. Correlation analysis, confirmatory factor analysis (CFA), mediation analysis, and moderation were performed.

## 4 Results and discussion

### 4.1 Descriptive statistics

Researchers gathered the responses of 420 consumers through online and self-attempted questionnaires. The gender of respondents is divided into two categories i.e. male and female. Male respondents represented 52.1% of the sample whereas female respondents were 47.9%. The age group from 18-31 years represented the majority of the respondents i.e. 94.0% followed by the age group of 32–44 years (5.2 %), and 45–57 (0.8%). The majority of the respondents were having a graduation degree (49.3%) followed by a Master's degree (43.1%), intermediate (6.4%), and matriculation (1.2%).

### 4.2 Confirmatory factor analysis (CFA)

**Figure 1:**
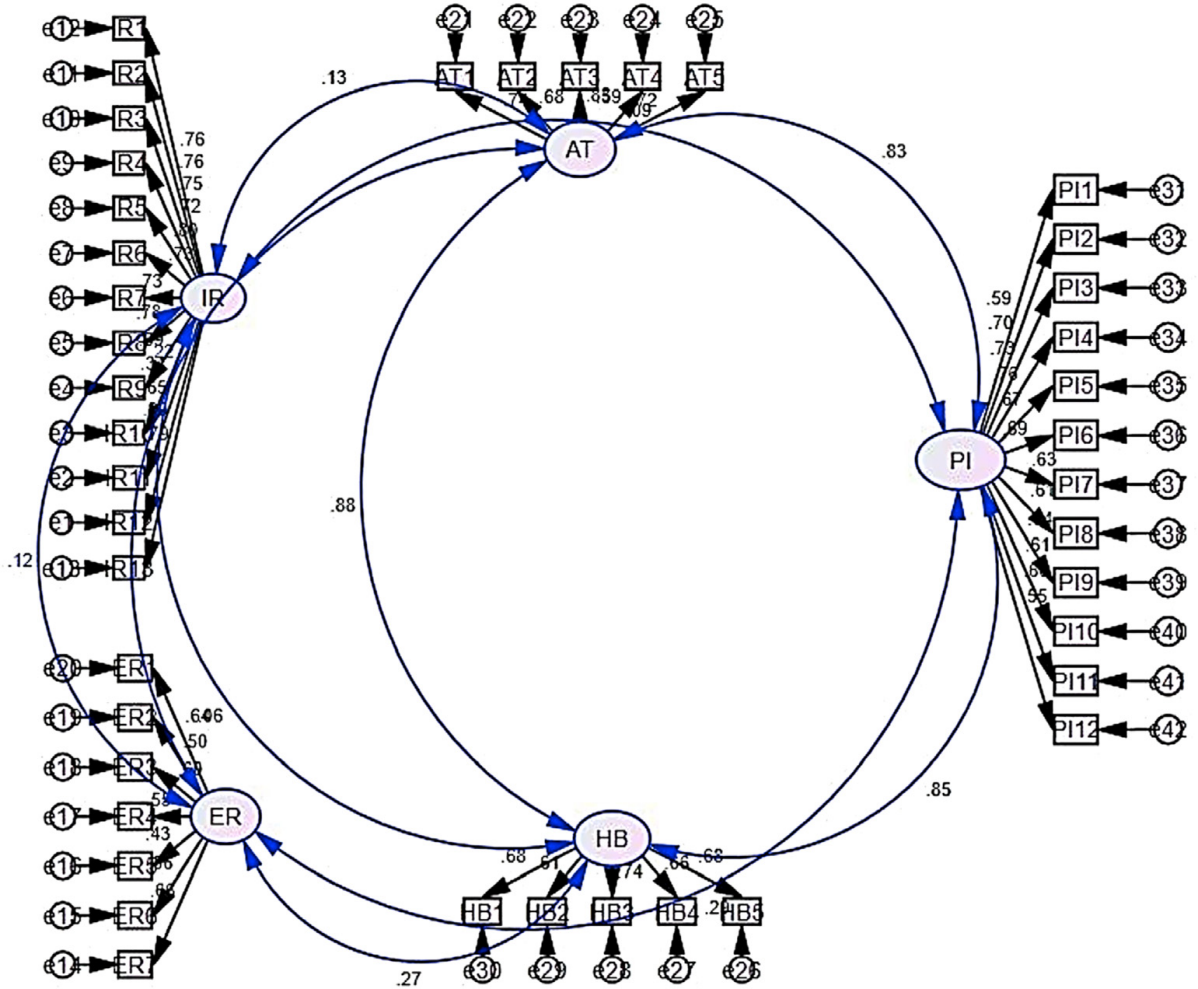
Factor loadings.

We conducted confirmatory factor analyses (CFA) for all the variables and the items were excluded from the model that had low factor loadings. CFA estimated the factor structure of the data set. Model Fit in CFA indicated how to fit the proposed model accounted for the correlations between variables. The results are provided in the figure given below (see Figure 1):

At this stage of specification search, the generated modification indices were examined. These indices offer an appraisal of the alteration in χ2 in case a correlation is detected between the error terms. It also provides an additional structural path. But these alterations required theoretical justification and these changes comprised of elimination of observed variables for fit I improvement. The specification search is a process consisting of three stages: the recursive process of detection, re-specification, and re-estimation is the third stage. All the process is repeated until the required and acceptable fit of the model is acquired. MacCallum (1986) suggested the recursive method of assessment of prespecified model by the process of step-wise elimination of observed variables (items) at one time and then again assessment of the re-specified model.

### 4.3 Analysis of measurement model

Researchers analyzed two ways as per SEM requirements i.e. model fitness, validity, reliability, and measurement of the model. Due to a misfit in our model that indicated low factor loading, we eliminated fourteen items for the improvement of reliability and model fit. Purchase Intention (PI1, PI7, PI8, PI9, PI10, PI11, PI12) after eliminating this model fitness, was improved. In the final measurement model, validity and reliability were measured.

### 4.4 Validity analysis

After eliminating the required items, the analysis was run. The results are in the table given below (see Table 1):

**Table 1:** Validity analysis.

Variables	CR	AVE	MSV	Intrinsic Religiosity	Extrinsic Religiosity	Attitude	Hedonic Benefit	Purchase Intention
Intrinsic Religiosity	0.934	0.527	0.316	0.726				
Extrinsic Religiosity	0.705	0.549	0.474	0.037	0.741			
Attitude	0.837	0.509	0.471	0.127	0.23	0.845		
Hedonic Benefit	0.772	0.53	0.368	0.099	0.229	0.826	0.795	
Purchase Intention	0.844	0.52	0.471	0.120∗	0.271	0.745	0.721	0.721

**Figure 2:**
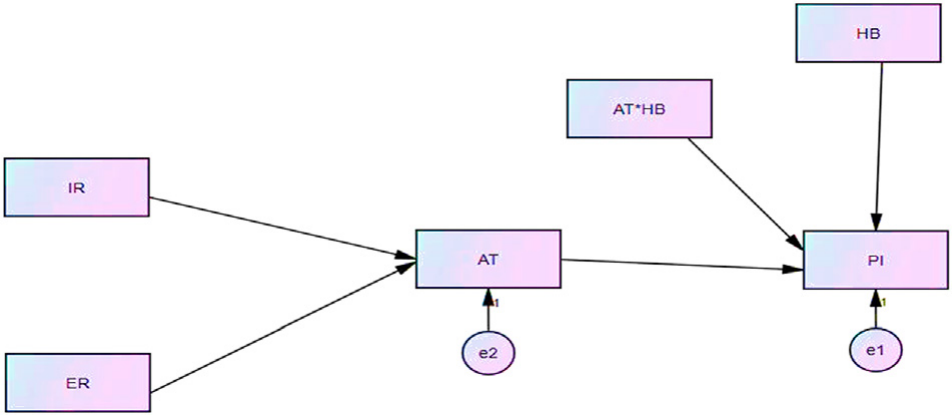
Structural equation modeling (SEM).

The results showed that all variables' CR values were greater than 0.7 and all AVE values were greater than 0.5 which means that they were acceptable according to the range of CR and AVE (see Figure 2).

### 4.5 Structural Equation Modeling (SEM)

The results showed that intrinsic religiosity had positive and significant relationship with attitude (b = 0.108, p < 0.05), extrinsic religiosity had positive and significant relationship with attitude (b = 0.145, p < 0.01), and attitude∗ hedonic benefit had positive and significant relationship with purchase intention (b = 0.207, p < 0.01). Hedonic benefit had positive and significant relationship with purchase intention (b = 0.218, p < 0.01), and attitude had positive and significant relationship with purchase intention (b = 0.407, p < 0.01). According to the results, hedonic benefit moderated the relationship between attitude and purchase intention, intrinsic religiosity had a direct impact on attitude and an indirect effect on purchase intention. Extrinsic religiosity had a direct impact on attitude and an indirect effect on purchase intention. The standardized regression weights are provided in the table given below (see Table 2):

**Table 2:** Standardized regression weights.

	Estimate	S.E.	C.R.	P	Label
AT < --- IR	.108	.045	2.419	.016	par_3
AT < --- ER	.145	.043	3.397	∗∗∗	par_4
PI < --- AT∗HB	.207	.006	5.501	∗∗∗	par_1
PI < --- HB	.218	.031	7.132	∗∗∗	par_2
PI < --- AT	.407	.032	12.780	∗∗∗	par_5

### 4.6 Indirect effects

The indirect effects were evaluated and the results are provided in the table given below (see Table 3):

**Table 3:** Indirect effects.

Indirect Path	Unstandardized Estimate	Lower	Upper	P-Value	Standardized Estimate
ER-- > AT-- > PI	0.059	0.018	0.127	0.002	0.081∗∗
IR-- > AT-- > PI	0.044	0.010	0.106	0.029	0.057∗

∗ Significant at 0.05 alpha level.

∗∗ Significant at 0.01 alpha level

The indirect effect of intrinsic religiosity on purchase intention by mediating the role of attitude is significant and positive (b = 0.057, p < 0.05), Indirect effect of extrinsic religiosity on purchase intention by mediating role of attitude is positive and significant (b = 0.081, p < 0.05) attitude (AT) is playing a mediating role between intrinsic religiosity (IR) and purchase intention and extrinsic religiosity (ER) and purchase intention (PI).

### 4.7 Acceptance/rejection of hypotheses

Based on the results of Structural Equation Modeling (SEM), the researchers accepted the hypotheses developed for testing by the current study. The following table provides information regarding the decision of acceptance or rejection of the hypotheses (see Table 4):

**Table 4:** Results of hypothesis testing.

Serial No.	Hypothesis	Statistical Results	Decision
H_1_	Intrinsic religiosity has positive and significant relationship with attitude.	b = 0.108 p < 0.05	Accepted
H_2_	Extrinsic religiosity has positive and significant relationship with attitude.	b = 0.145 p < 0.05	Accepted
H_3_	Attitude has positive and significant relationship with purchase intentions.	b = 0.407 p < 0.05	Accepted
H_4_	Hedonic benefit has positive and significant relationship with purchase intentions.	b = 0.218 p < 0.05	Accepted
H_5_	Attitude mediates the relationship between intrinsic religiosity and purchase intentions.	b = 0.057 p < 0.05	Accepted
H_6_	Attitude mediates the relationship between extrinsic religiosity and purchase intentions.	b = 0.081 p < 0.05	Accepted
H_7_	Hedonic benefit moderates the relationship between attitude and purchase intentions.	b = 0.207 p < 0.05	Accepted

## 5 Discussion of findings

The study was aimed at examining the religiosity model while studying the purchase intentions of counterfeit products. This study investigated the role of attitude, hedonic benefits, intrinsic religiosity, and extrinsic religiosity that determined the purchase intention to purchase counterfeit products. It was found that both intrinsic religiosity and extrinsic religiosity positively affected the attitude of consumers in developing their purchase intentions towards buying counterfeit products. These findings authenticated the findings of Bupalan et al. (2019) and are in alignment with the findings of Mortimer et al. (2020) and Nurhayati and Hendar (2019).

The current study found a positive and significant relationship between intrinsic religiosity and attitude, a positive and significant relationship between extrinsic religiosity with attitude, and a positive and significant relationship of attitude with purchase intentions. All three findings are similar to the findings of Rahman et al. (2015). It was found that hedonic benefit had a positive and significant relationship with purchase intentions which is similar to the findings of Lee and Yun (2015). This study found that attitude mediated the relationship between intrinsic religiosity and purchase intentions which is similar to the findings of Garg and Joshi (2018). It was found that attitude mediated the relationship between extrinsic religiosity and purchase intentions which confirmed the empirical findings of Pace (2014). The current study found that hedonic benefit moderated the relationship between attitude and purchase intentions which authenticated the findings of Das et al. (2018).

This study has advanced the knowledge in the field as required by the previous studies of Bupalan et al. (2019), Nurhayati and Hendar (2019), and Mortimer et al. (2020). The producers can use this finding to make an appropriate plan and execute them in reaping optimum benefits for themselves. However, fraudulent intentions need to be eliminated. As the hedonic benefit moderated this relationship, it can be asserted that adding this aspect i.e. providing hedonic benefits to the potential consumers would add extra advantage for the producers. Moreover, significant mediation of attitude towards creating purchase intentions reveals that the producers have to address the issues regarding attitudes of the consumers through which ultimate purchase intentions would be created. Regarding counterfeit products, community factors and a consumer's personal inspirations play an important role in driving his/her intentions to purchase the counterfeit products. Consequently, all the hypotheses developed by the researchers are accepted.

## 6 Conclusions

The current study examined the impact of intrinsic and extrinsic religiosity with the mediating role of attitude and moderating role of hedonic benefits towards purchase intentions of counterfeit products. As this study provided a statistically significant link between religiosity and purchase intention of counterfeit products, managers and academic scholars can use these findings to advance this discussion. This reasoning is provided based on purchasing of the counterfeit products and why the consumers prefer the counterfeit products. However, fraudulent (counterfeit) intentions need to be eliminated. We solemnly discourage the counterfeit products/services intended to deceive the consumers/customers. Based on results and findings it can be safely concluded that religiosity plays important role in inducing and instigating purchase intentions among the consumers. Therefore, the corporations should consider religiosity while producing and advertising their products or services which are not fraudulent.

## 7 Recommendations

This study carries some limitations that might be used as possible avenues for future research directions. This study recommends the corporations consider religiosity as an important factor while producing and advertising their products or services. In terms of academic discussion, the current study used intrinsic and extrinsic religiosity. However, it is recommended to use five dimensions of religiosity in future research. Those dimensions include beliefs, rituals, intellectuals, experiences, and consequences. This would provide an elaborated model of religiosity and the results would generate fruitful findings to be taken as a guideline by the practitioners and food for thought by the scholars.

## Declarations

### Author contribution statement

M. Munir, A.Y. Yaakop, H.M. Hafeez and M.M. Faisal:Conceived and designed the experiments; Performed the experiments; Analyzed and interpreted the data; Contributed reagents, materials, analysis tools or data; Wrote the paper.

### Funding statement

This research did not receive any specific grant from funding agencies in the public, commercial, or not-for-profit sectors.

### Data availability statement

Data will be made available on request.

### Declaration of interests statement

The authors declare no conflict of interest.

### Additional information

No additional information is available for this paper.
